# Supplementary material for: Ultrafast self-heating synthesis of robust heterogeneous nanocarbides for high current density hydrogen evolution reaction
Source: Nat Commun. 2022 Jun 9;13:3338. doi: 10.1038/s41467-022-31077-x (PMC9184596; doi:10.1038/s41467-022-31077-x)
Supplement: Supplementary file 1 — Supplementary Information [file 41467_2022_31077_MOESM1_ESM.pdf]

Supplementary Information

Ultrafast self-heating synthesis of robust heterogeneous nanocarbides for high current density hydrogen evolution reaction

Chenyu Li[1‡], Zhijie Wang[2‡], Mingda Liu[1‡], Enze Wang[1], Bolun Wang[1], Longlong Xu[1], Kaili Jiang[3], Shoushan Fan[3], Yinghui Sun[4*], Jia Li[2*] & Kai Liu[1*]

[1] State Key Laboratory of New Ceramics and Fine Processing, School of Materials Science and Engineering, Tsinghua University, Beijing 100084, China.

[2] Shenzhen Geim Graphene Center and Institute of Materials Research, Tsinghua Shenzhen International Graduate School, Tsinghua University, Shenzhen 518055, P. R. China.

[3] Department of Physics and Tsinghua-Foxconn Nanotechnology Research Center, Tsinghua University, Beijing, 100084, China.

[4] Beijing Key Laboratory for Magneto-Photoelectrical Composite and Interface Science, School of Mathematics and Physics, University of Science and Technology Beijing, Beijing, 100083, China.

[‡] These authors contributed equally to this work.

[*] Corresponding authors. Emails: liuk@tsinghua.edu.cn (K.L.); li.jia@sz.tsinghua.edu.cn (J.L.); yhsun@ustb.edu.cn (Y.S.)

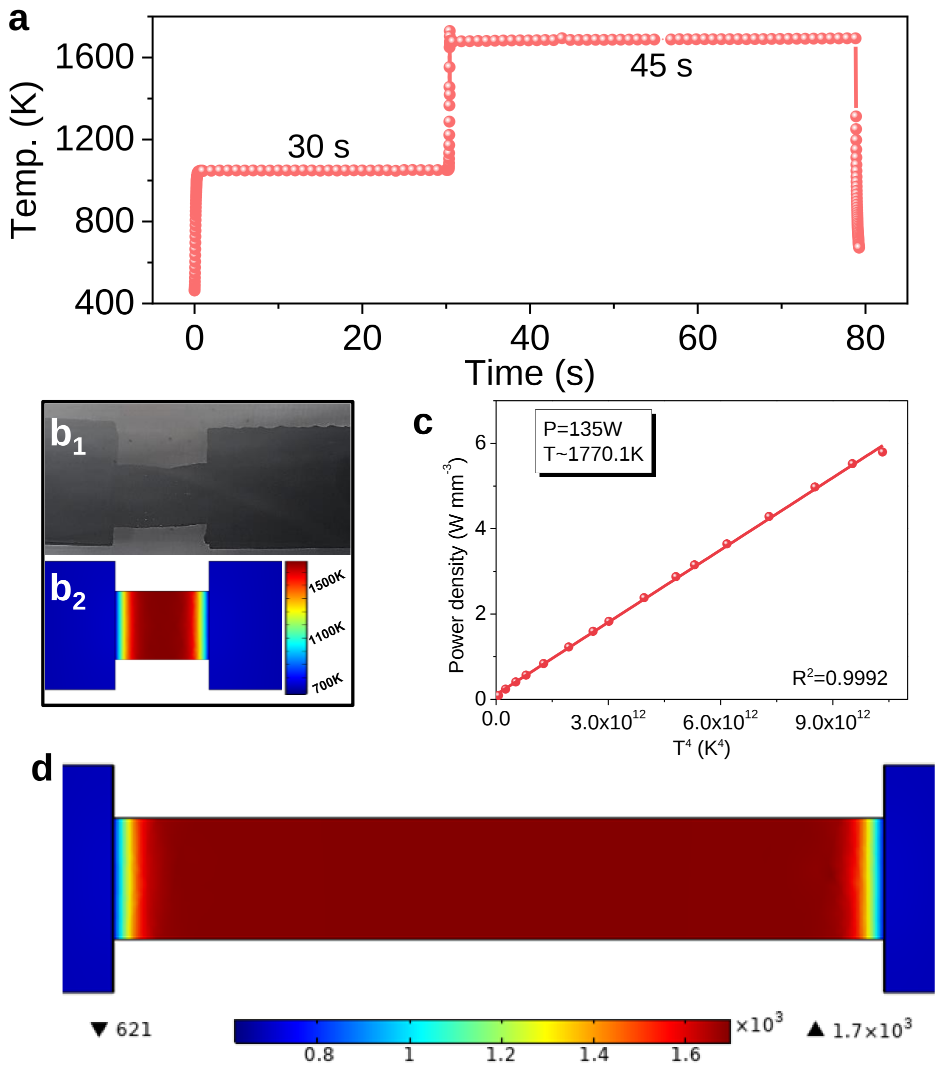

Supplementary Fig. 1 | Ultrafast self-heating synthesis. a Temperature control curve of the self-heating process, including a heating process of 30 s at 30 W followed by the other process of 45 s at 135 W. **b_1** Optical pictures of CNT film (15mm × 8mm) under 0W. **b_2** Simulated temperature distribution at ~1700 K. **c** Power density-T^4 curve of CNT film (15 mm × 8 mm) obtained using an infrared thermometer presented a good linear relationship, indicating that the actual temperature was ~1190/1770 K at ~1.244/5.659 W mm^{-3}. **d** Simulated temperature distribution of a 50mm × 8 mm CNT film.

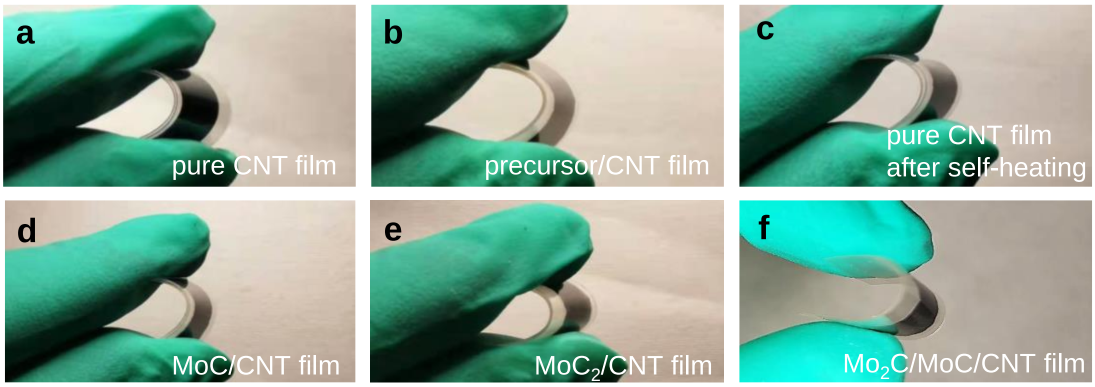

Supplementary Fig. 2 | Optical images of samples. a,b Optical images showing the good flexibility of a pure CNT film and a precursor/CNT film before a self-heating process. **c-f** Optical images of a pure CNT film, MoC/CNT film, Mo_2C/CNT film, and Mo_2C/MoC/CNT film, respectively, after the self-heating process. All of the films keep flexible before or after the treatment.

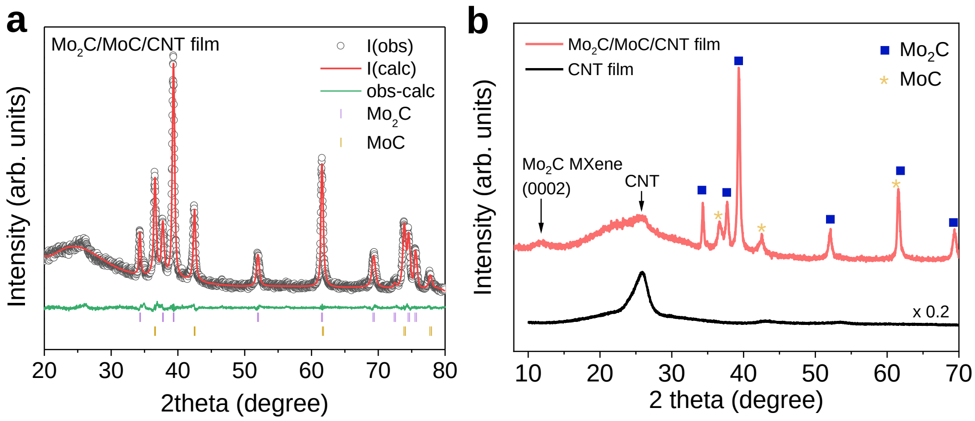

Supplementary Fig. 3 | XRD analysis of Mo_2C/MoC/CNT films. a XRD peak fitting of Mo_2C/MoC/CNT film by Le Bail and Rietveld refinement in the analysis software GSAS. R_{wp}=7.1%. **b** XRD patterns of a Mo_2C/MoC/CNT film and a pure CNT film.

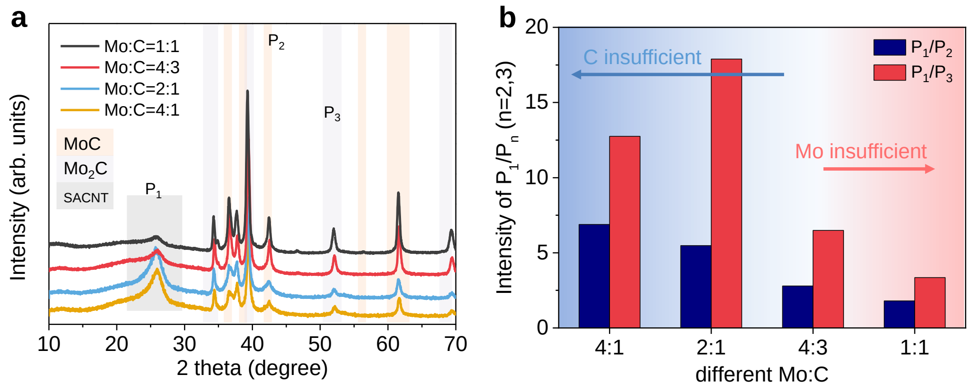

Supplementary Fig. 4 | XRD characterizations of Mo_2C/MoC/CNT films synthesized with different Mo:C ratios in precursor. a XRD patterns of Mo_2C/MoC/CNT film synthesized with different Mo:C ratios in precursor. P_1, P_2, and P_3 belong to CNT (002), MoC (200), and Mo_2C (102), respectively. **b** Intensity of P_1/P_2 and P_1/P_3 in different samples.

The adjusted factors are the ratio of ammonium molybdate to glucose in precursor solution (denoted as Mo:C, without urea). All of the prepared composite films are composed of Mo_2C, MoC, and CNT, which are demonstrated by XRD patterns of samples with different Mo:C. The intensity ratio in XRD of CNT (002) (P_1) to MoC (200) (P_2) and CNT (002) (P_1) to Mo_2C (102) (P_3) gradually increases with the decrease of carbon content among different Mo:C samples, suggesting the decreased content of MoC and Mo_2C. The reason for the content change is that MoO_x from ammonium molybdate not only reacts with the carbon source but also evaporates meanwhile at high temperatures. A large amount of MoO_x volatilizing generates low content of Mo_xC ultimately for an insufficient carbon source. Besides, Supplementary Fig. 4 shows the change of the intensity ratio of P_2 and P_3 as well due to the different content of MoC

and Mo_2C. In our method, MoC further converts into Mo_2C on the condition of high temperature and reduction atmosphere while the content of Mo_2C is almost the same when the heating time and temperature are united, which suggests that the initial content of MoC will determine the final MoC:Mo_2C. Therefore, Mo:C in the precursor is an essential factor for regulating final MoC:Mo_2C because MoC is derived from those MoO_x reacting with carbon sources.

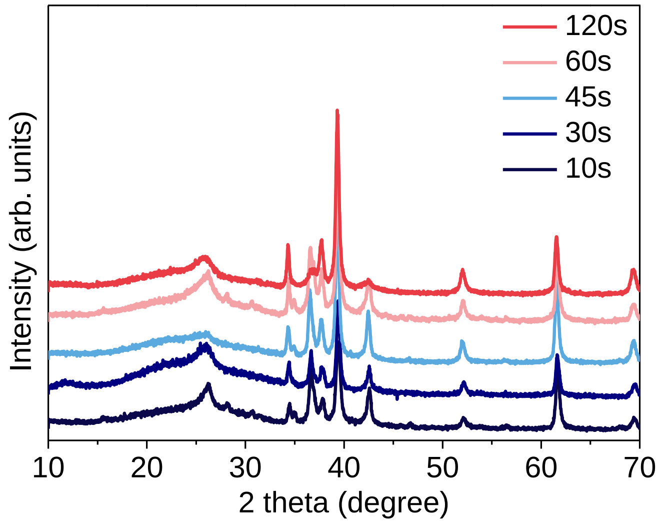

Supplementary Fig. 5 | XRD patterns of Mo_2C/MoC/CNT films synthesized with different heating times. The heating time ranges from 10 to 120 s and the heating power is 135W.

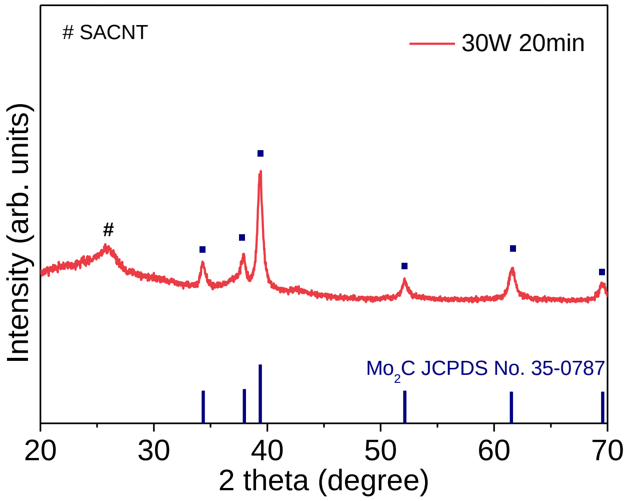

Supplementary Fig. 6 | XRD patterns of a sample synthesized with a long reaction time. The heating time is 20 mins and the heating power is 30 W.

When the heating time is long enough, only Mo_2C phase exists rather than the hybrid Mo_2C/MoC, which concludes that a short reaction time is crucial for synthesizing Mo_2C/MoC heterostructure. We find that if the heating time is 20 minutes even at a low power of 30 W, the composition will completely convert into Mo_2C, as shown in Supplementary Fig. 6. As a result, it is difficult for traditional methods like furnace heating to form Mo_2C/MoC heterostructure owing to an inevitable long-time heating, heat preservation, and cooling process.

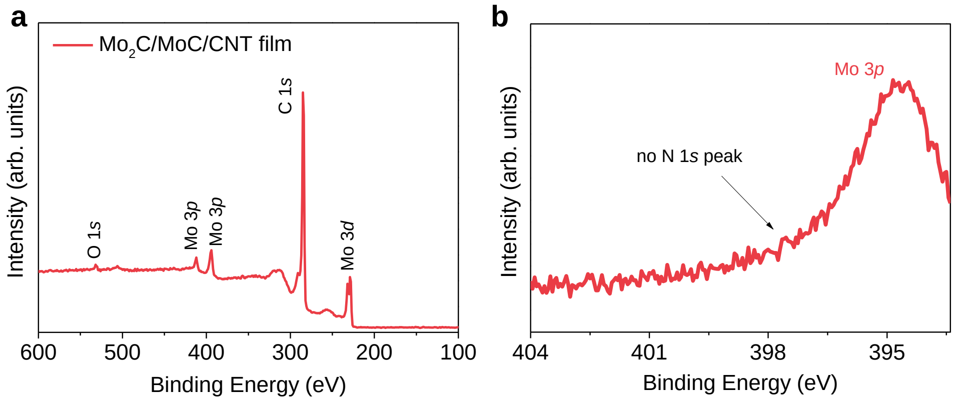

Supplementary Fig. 7 | XPS spectra of Mo_2C/MoC/CNT films. a Full spectra. **b** N 1s range.

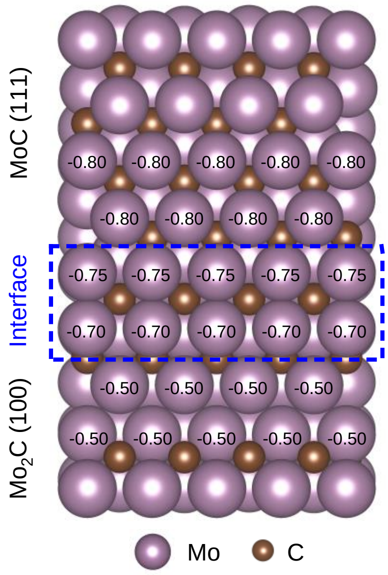

Supplementary Fig. 8 | DFT calculations of charge transfer at the Mo_2C/MoC interface. It indicates that electrons transfer from Mo_2C to MoC regions.

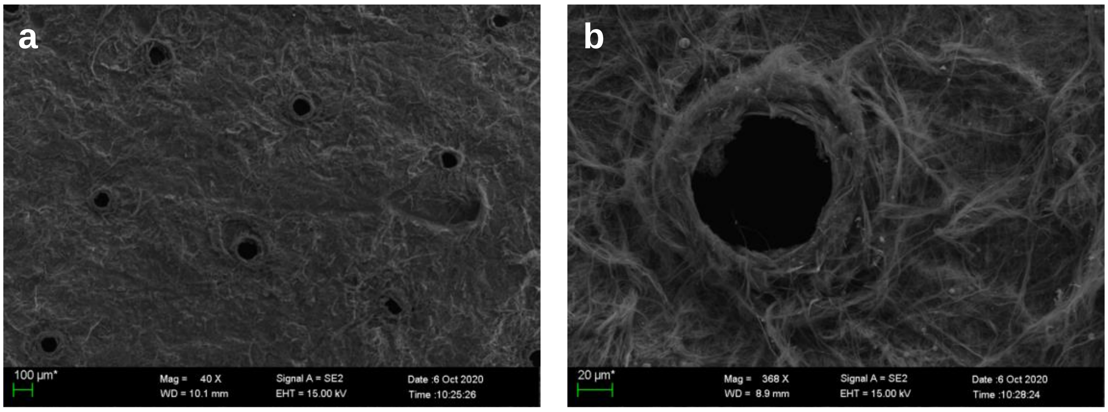

Supplementary Fig. 9 | SEM images of micropores. a,b SEM images of the micropores drilled by laser in Mo_2C/MoC/CNT film under different magnifications. The micropores serve as tunnels for H_2 release during HER and enhance the stability of electrodes.[1]

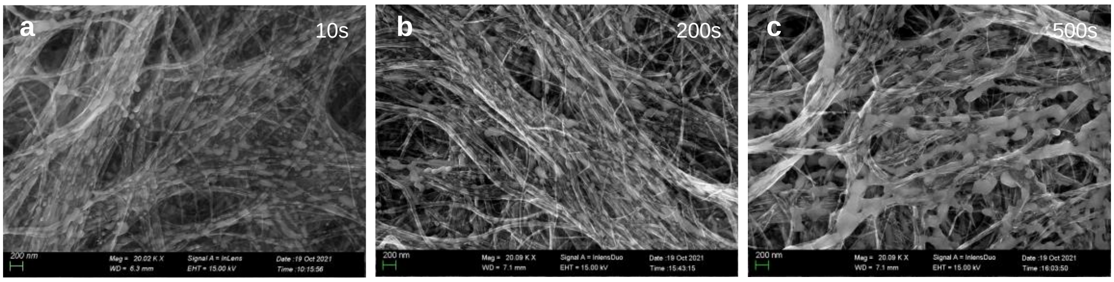

Supplementary Fig. 10 | SEM images of Mo_2C/MoC/CNT films synthesized at different heating ramp rates. The heating ramp time of as-prepared Mo_2C/MoC/CNT films for both heating steps from room temperature to ~ 1100 K (30 W) and from ~ 1100 to ~1770 K (135 W) is **a** 10 s, **b** 200 s, and **c** 500 s, respectively.

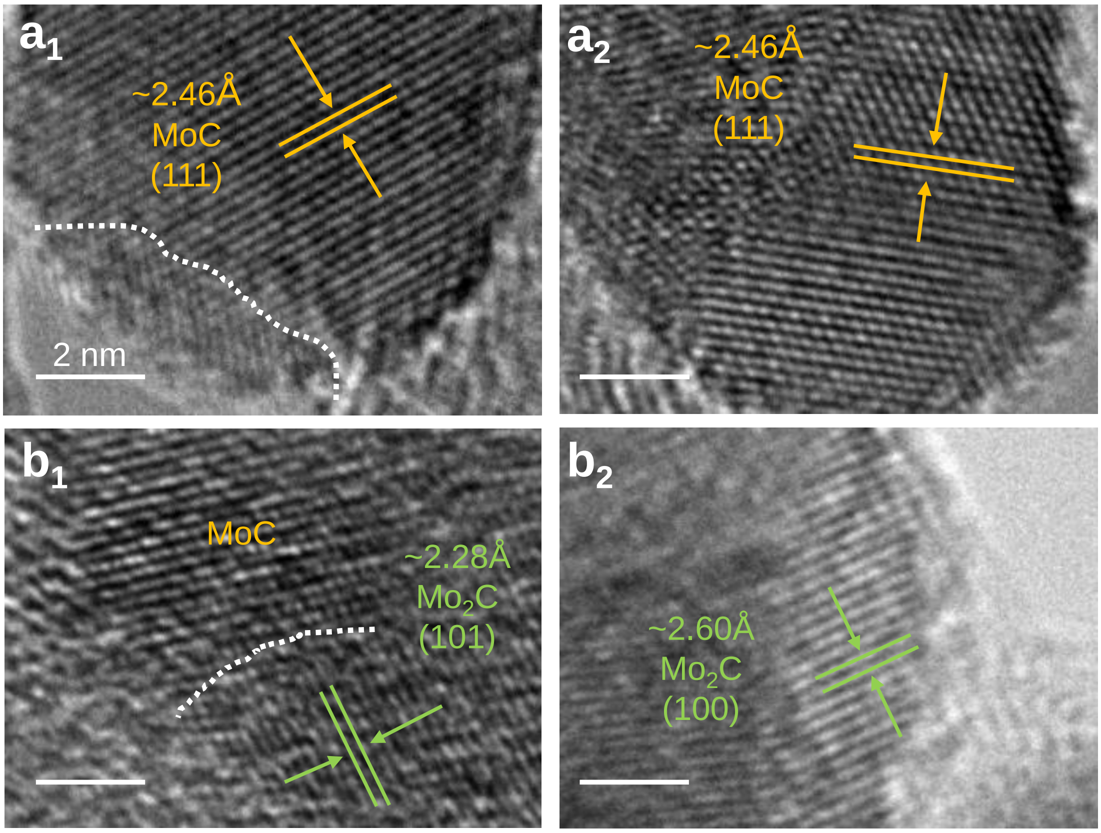

Supplementary Fig. 11 | HRTEM images of Mo_2C/MoC/CNT films. a_1,a_2 Lattice fringes of ~2.46Å, which belongs to MoC (111) plane. **b_1,b_2** Lattice fringes of ~2.28Å and ~2.60Å, which belong to Mo_2C (101) plane and (100) plane. The white lines in **a_1** and **b_1** indicate the interface between hybrid domains.

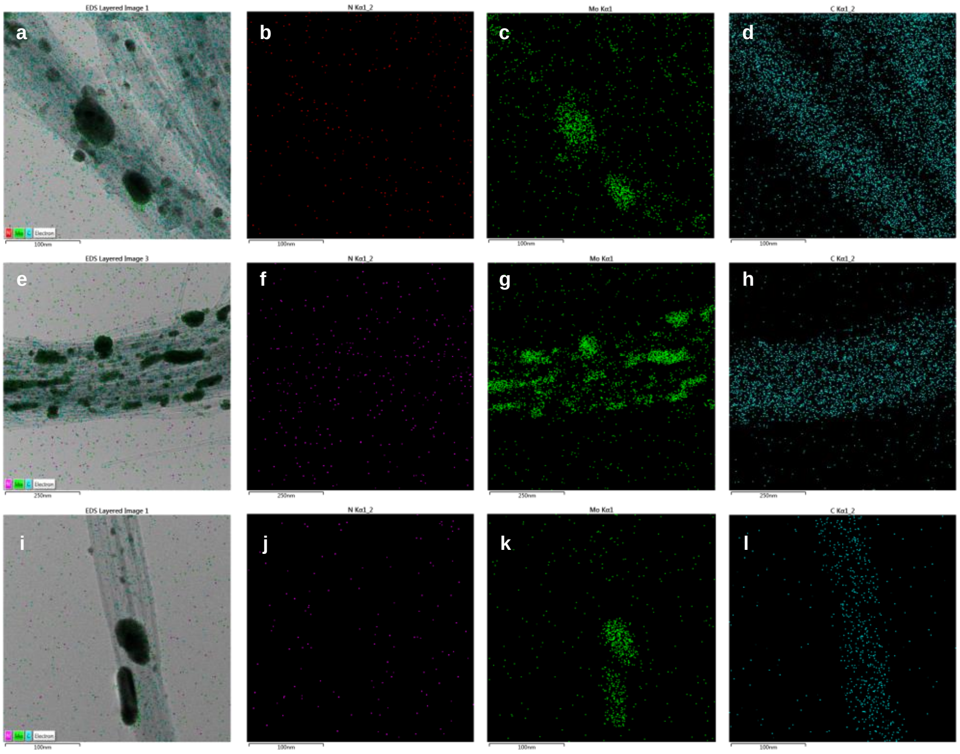

Supplementary Fig. 12 | EDS mapping analysis. a,**e**,**i** EDS mapping of N, Mo, and C element, respectively, for Mo_2C/MoC/CNT films. **b**,**f**,**j** N signals, indicating that N element does not exist in Mo_2C/MoC/CNT films. **c**,**g**,**k** Mo signals (green dots). **d**,**h**,**l** C signals (blue dots).

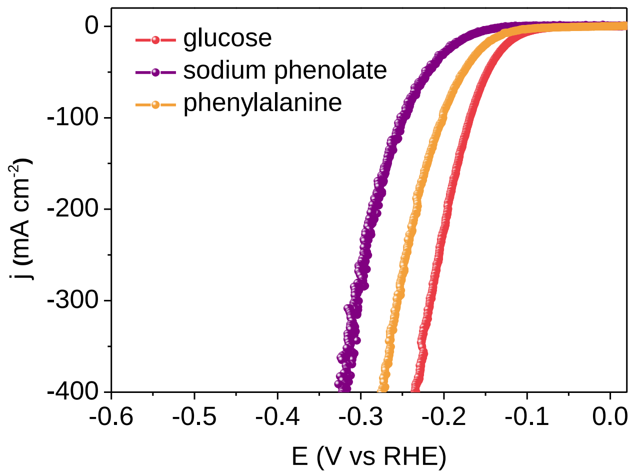

Supplementary Fig. 13 | Polarization curves of samples synthesized with different C sources. These samples were synthesized using glucose, sodium phenolate, and phenylalanine as C sources in the self-heating method.

We involve different small molecules as carbon sources including glucose, sodium phenolate, and phenylalanine. Glucose reaches the smallest overpotential and is most suitable for our rapid self-heating method, which may due to the higher activity of C atoms in glucose making C-C bonds easier to break and then Mo-C bonds form within a short time.

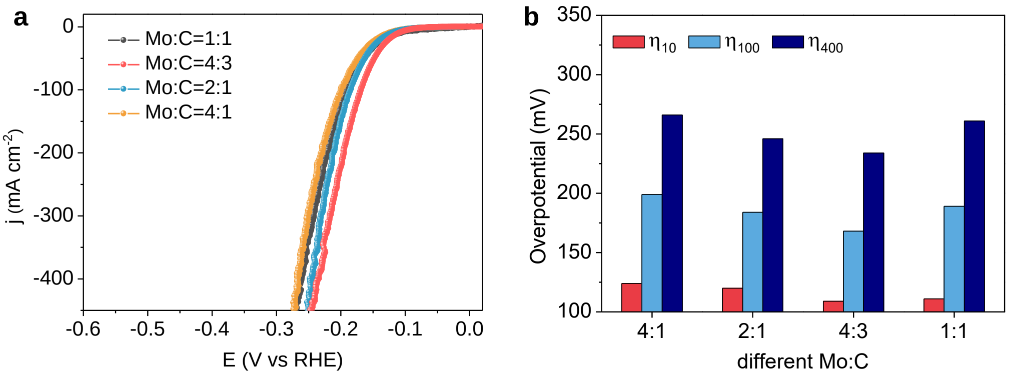

Supplementary Fig. 14 | Polarization curves of samples synthesized with different Mo:C ratios in precursor. a Polarization curves of samples synthesized with Mo:C = 1:1, 4:3, 2:1, and 4:1 in precursor, measured in 1M KOH. **b** Overpotential at 10, 100, and 400 mA cm^{-2} for samples synthesized with Mo:C = 1:1, 4:3, 2:1, and 4:1.

The ratio of Mo to C in the precursor will regulate the ratio and total load of two Mo-C phases ultimately (MoC and Mo_2C). The hybrid catalysts reach lower HER overpotential when Mo:C is ~4:3 through the LSV test. When Mo:C is large, insufficient C source is unable to react with all of Mo source timely, leading to a large amount of Mo evaporation and a small load of Mo_2C/MoC, with Mo_2C accounts for a relatively large proportion. When Mo:C is small, the excess C atoms and Mo_2C/MoC cover each other, preventing active sites in Mo_2C/MoC from exposing thoroughly.

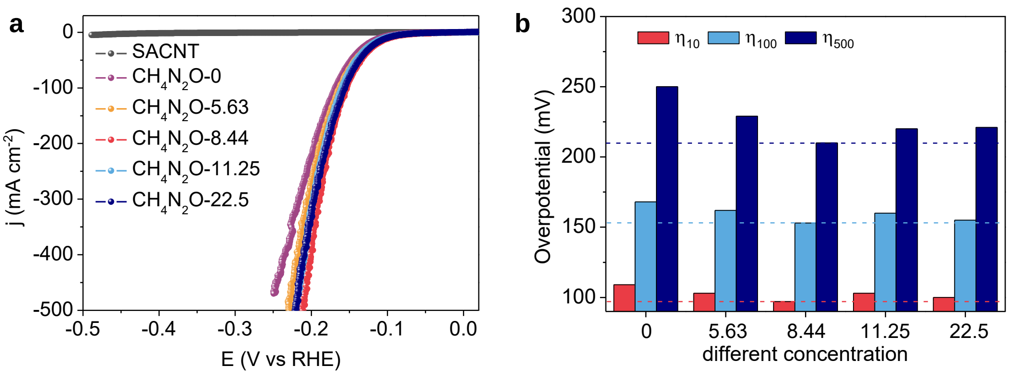

Supplementary Fig. 15 | Polarization curves of samples synthesized with different contents of CH_4N_2O in precursor. **a** Polarization curves (measured in 1M KOH) of samples synthesized with different amounts of CH_4N_2O by 0, 5.63, 8.44, 11.25, and 22.5 mg in precursor, which represent a ratio of glucose to urea as 0, 10:1, 20:3, 5:1, 5:2 in our experiment. **b** Overpotential at 10, 100, and 500 mA cm^{-2} for samples with different amounts of CH_4N_2O.

Adding a light content of urea improves the HER performance to a certain extent, and the smallest overpotential can be reached when glucose: urea is ~20:3.

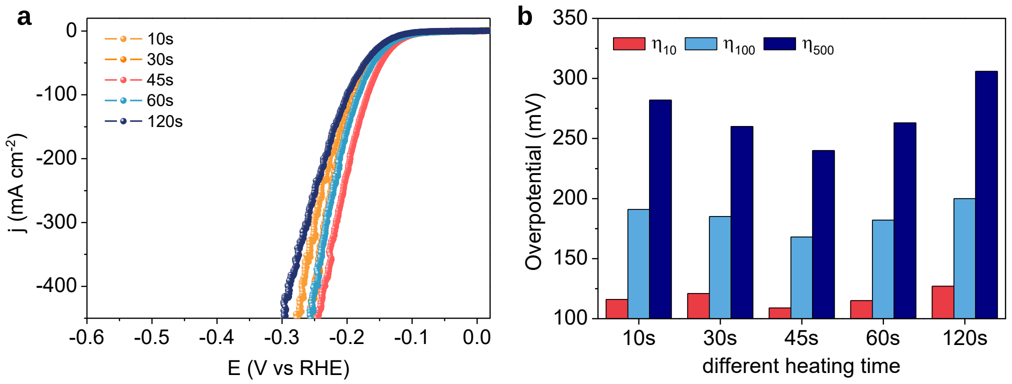

Supplementary Fig. 16 | Polarization curves of samples synthesized with different heating time. a Polarization curves for samples synthesized with different heating time ranging from 10s to 120s at 135W, measured in 1M KOH. **b** Overpotentials at 10, 100, and 500 mA cm^{-2} for samples synthesized with different heating time at 135W.

MoC is the main phase with a short self-heating time while Mo_2C becomes the main phase with a long self-heating time at high temperature, both of which lead to the reduction of interfaces between hybrid domains.

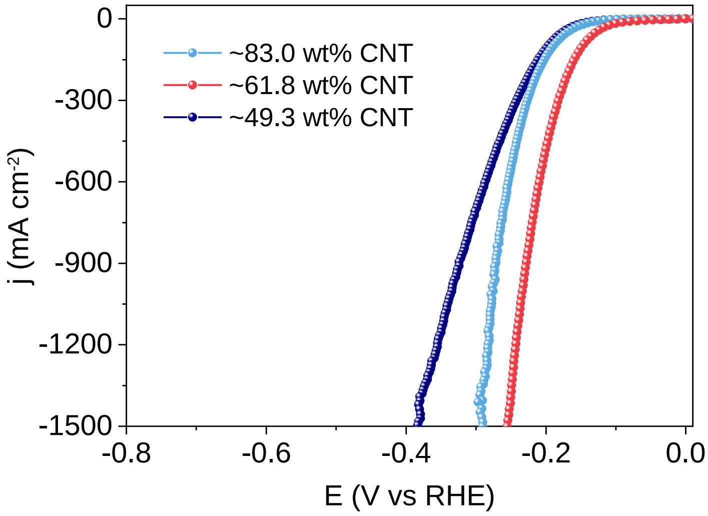

Supplementary Fig. 17 | Polarization curves of Mo_2C/MoC/CNT films with different CNT wt%. The CNT wt% ranges from 49.3 to 83.0 wt%.

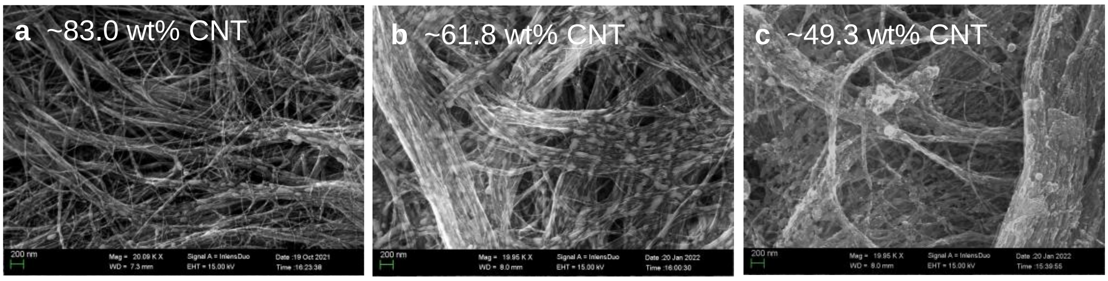

Supplementary Fig. 18 | SEM images of Mo_2C/MoC/CNT films with different CNT wt%. The CNT wt% in the Mo_2C/MoC/CNT samples is **a** 83.0%, **b** 61.8%, and **c** 49.3%, respectively.

The sample with the moderate CNT wt% (~ 61.8 wt% CNT and ~ 38.2 wt% Mo_2C/MoC) possesses the best HER activity. It exhibits no apparent agglomeration of nanoparticles and thus has abundant Mo_2C/MoC interfacial area. More or less content of Mo_2C/MoC will lead to poorer HER activity. When the content of CNT is high, the

content of MoC or Mo_2C is very few and thereby the total Mo_2C/MoC interfacial area becomes very limited. And for the samples with a low content of CNT, Mo_2C/MoC will agglomerate during the self-heating process, also reducing the total Mo_2C/MoC interfacial area. This result suggests that an appropriate CNT wt% is needed to increase Mo_2C/MoC interfacial area for better HER.

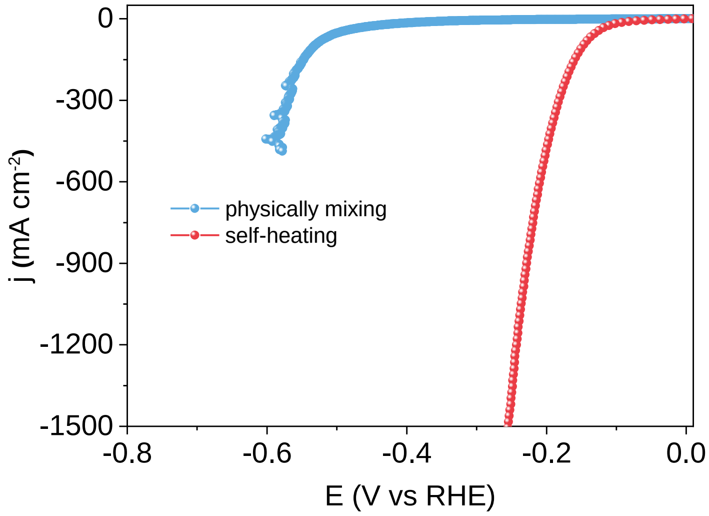

Supplementary Fig. 19 | Polarization curves of Mo_2C/MoC/CNT films prepared by different methods. The physically mixed Mo_2C/MoC/CNT film is made by loading mixed MoC and Mo_2C powders onto the CNT film.

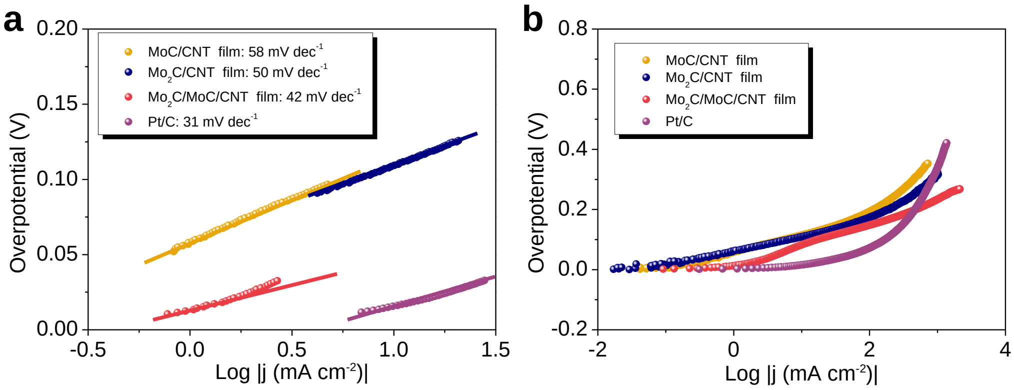

Supplementary Fig. 20 | Tafel plots of Mo_2C/MoC/CNT film, Mo_2C/CNT film, MoC/CNT film, and Pt/C. a Potential range of 0.01 to 0.13 V. **b** Potential range of 0 to 0.42 V.

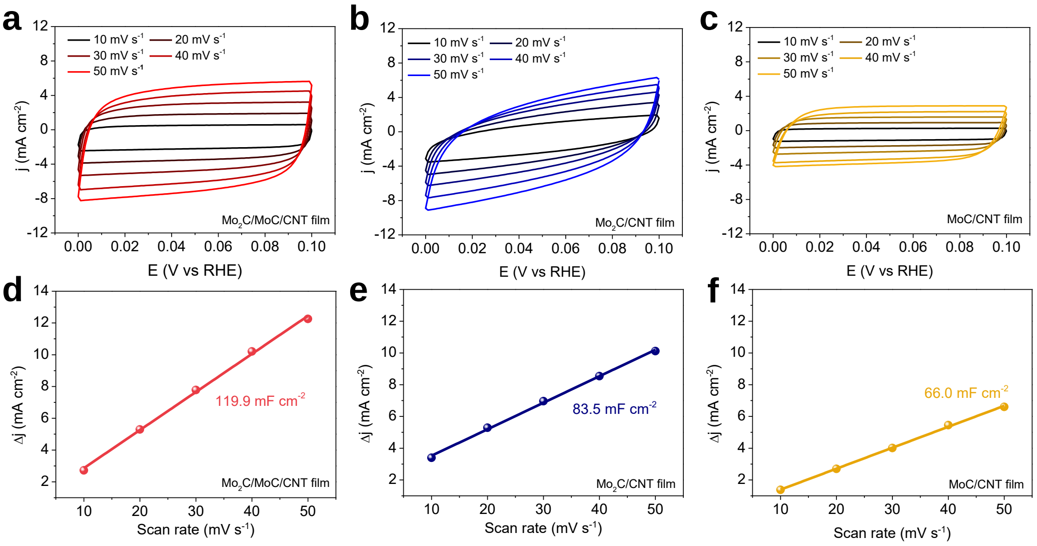

Supplementary Fig. 21 | Cyclic voltammograms (CVs) used to determine the electrochemical C_{dl}. a,**d** Mo_2C/MoC/CNT film. **b**,**e** Mo_2C/CNT film. **c**,**f** MoC/CNT film. All of the CV curves were measured at different scan rates in 1M KOH.

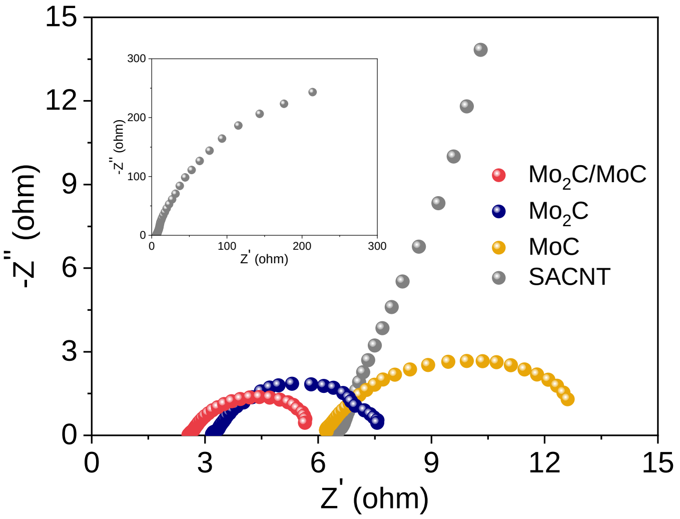

Supplementary Fig. 22 | Nyquist plots of different materials. The plots were obtained at a potential of -150 mV versus RHE in 1M KOH.

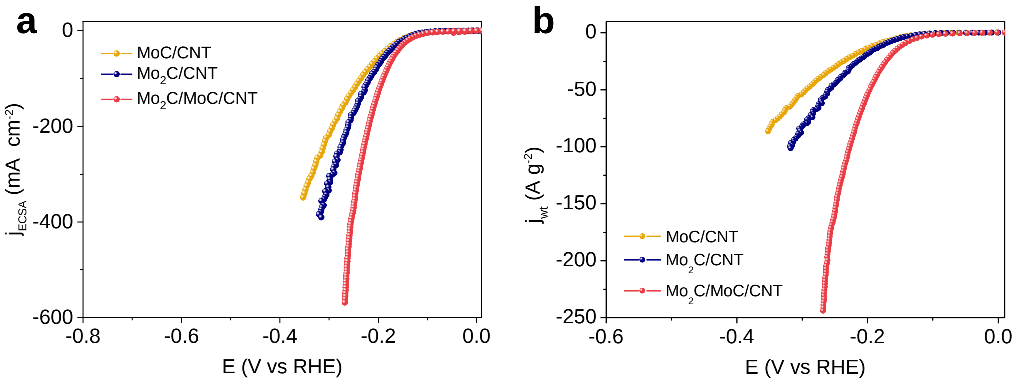

Supplementary Fig. 23 | Normalized polarization curves of Mo_2C/MoC/CNT film, Mo_2C/CNT film, and MoC/CNT film. They are normalized by **a** ECSA and **b** mass of active materials (i.e., Mo_2C/MoC, Mo_2C, and MoC, respectively).

The electrochemical surface area (ECSA) of each sample can be evaluated from the double-layer capacitance (C_{dl}) according to

$$\mathrm{ECSA}=\frac{C_{dl}}{C_s},$$

where C_s is the specific capacitance of the sample or the capacitance of an atomically smooth planar surface of the material per unit area under the same condition. C_s for a flat surface is generally found to be in the range of 20-60 μF cm^{-2},[2, 3] and the value of 40 μF cm^{-2} is used in this work to calculate the ECSA.

The ECSAs of Mo_2C/MoC/CNT film, Mo_2C/CNT film, and MoC/CNT film are calculated to be 2998, 2088, and 1650 cm^2, respectively, according to previous CV tests. The mass of the active materials, i.e., Mo_2C/MoC on the Mo_2C/MoC/CNT film, Mo_2C on the Mo_2C/CNT film, and MoC on the MoC/CNT film, are 2.1, 2.4, and 2.0 mg, respectively, which have only a little difference. The polarization curves of the three films normalized to ECSA and mass of active materials are replotted in Supplementary Fig. 23a and Fig. 23b, respectively.

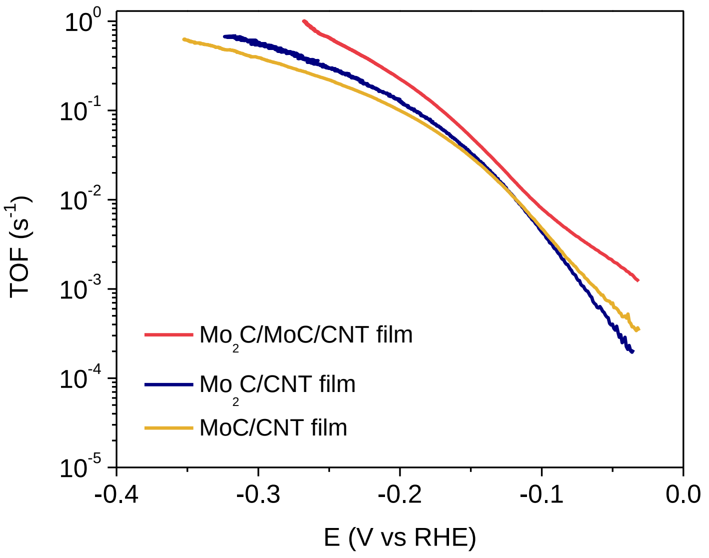

Supplementary Fig. 24 | Turnover frequency per surface Mo atom (TOF_{Mo}). The TOF of Mo_2C/MoC/CNT film, Mo_2C/CNT film, and MoC/CNT film is 0.65, 0.30, and 0.22 s^{-1}, respectively.

The turnover frequency (TOF) is calculated by the following equation:[2,4]

$$TOF(s^{-1}) = \frac{\text{number of total hydrogen turnovers/cm}^2}{\text{number of active of sites/cm}^2} = \frac{|J|\ (\text{mA cm}^{-2})}{n \times 1000 \times N \times ECSA \times (1.602 \times 10^{-19} C)} = \frac{3.12 \times 10^{15} \times |J|}{N \times ECSA},$$

where N is the density of active sites, n is the number of electrons involved in the reaction. The density of active sites can be calculated as follows,

$$N = \left(\frac{\text{Number of sites/unit cell}}{\text{Volume/unit cell}}\right)^{\frac{2}{3}}.$$

For MoC,

$$N_1 = \left(\frac{8 \text{ sites/unit cell}}{77.854 \text{ Å}^3\text{/unit cell}}\right)^{\frac{2}{3}} = 2.19 \times 10^{15} \text{ sites cm}^{-2}$$

For Mo_2C,

$$N_2=\left(\frac{4\ \text{sites/unit cell}}{37.459\ \text{Å}^3\text{/unit cell}}\right)^{\frac{2}{3}}=2.25\times10^{15}\ \text{sites cm}^{-2}$$

For Mo_2C/MoC,

$$N_3=xN_1+yN_2=2.21\times10^{15}\ \text{sites cm}^{-2},$$

where x=74%, y=26% according to the XRD peak fitting results.

At an overpotential of 250 mV, the TOF of $Mo_2C/MoC/CNT$ film, Mo_2C/CNT film, and MoC/CNT film is 0.65 s^{-1}, 0.30 s^{-1}, and 0.22 s^{-1}, respectively, which validates that $Mo_2C/MoC/CNT$ catalyst has higher intrinsic activity besides the larger ECSA. The increase of the intrinsic activity should be attributed to the abundant Mo_2C/MoC interfaces.

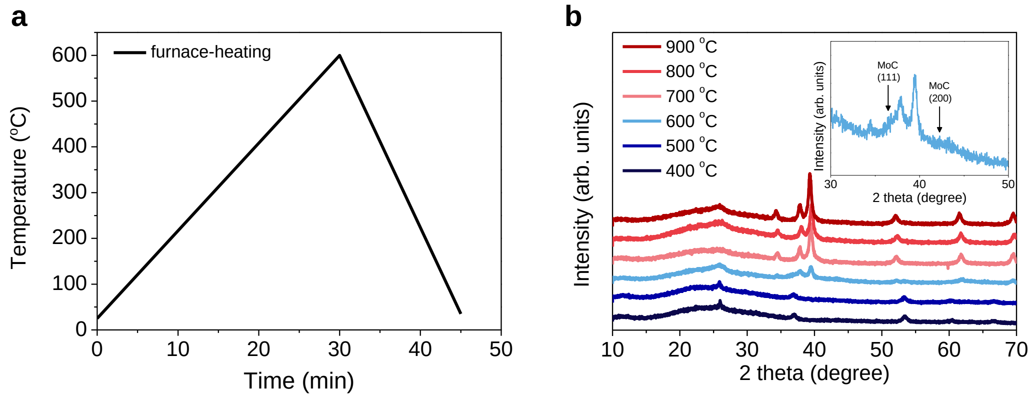

Supplementary Fig. 25 | Furnace-heating method. a Temperature rise and fall process in the furnace-heating method. **b** XRD patterns of different samples synthesized by the furnace-heating method at different temperatures.

The synthesis of the Mo_2C/MoC composite phase relies on a short reaction time at high temperature, otherwise, MoC will completely convert to Mo_2C. Therefore, it is difficult to synthesize the Mo_2C/MoC composite phase in a tube furnace for an inevitably long heating and cooling time. As shown in Supplementary Fig. 25, Mo_2C/MoC composite phase form only when heated to ~600°C, with incomplete carbonization from MoO_x to Mo_xC at lower temperature and excessive conversion from MoC to Mo_2C at a higher temperature. Thus in the electrochemical tests and characterization, we adopt 600°C as the reaction temperature for heating in the tube furnace.

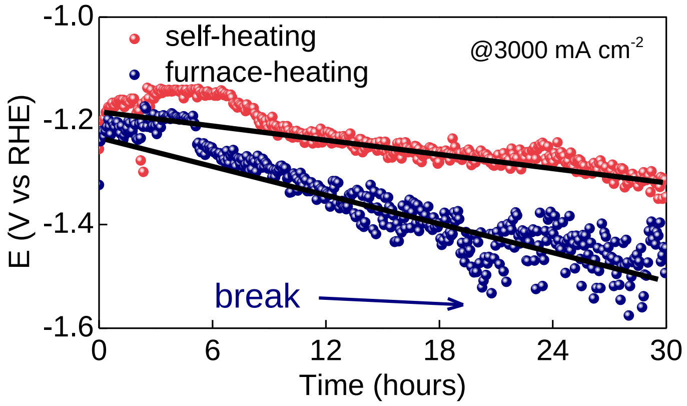

Supplementary Fig. 26 | Long-term HER stability tests of Mo_2C/MoC/CNT film and f-Mo_2C/MoC/CNT film. The tests were performed at 3000 mA cm^{-2} in 1M KOH by 30h-chronopotentiometry without iR compensation.

After working over 30 hours, f-Mo_2C/MoC/CNT film is about to break by the mechanical force from H_2 bubbles release, while Mo_2C/MoC/CNT film maintains the shape although the performance decay to some extent.

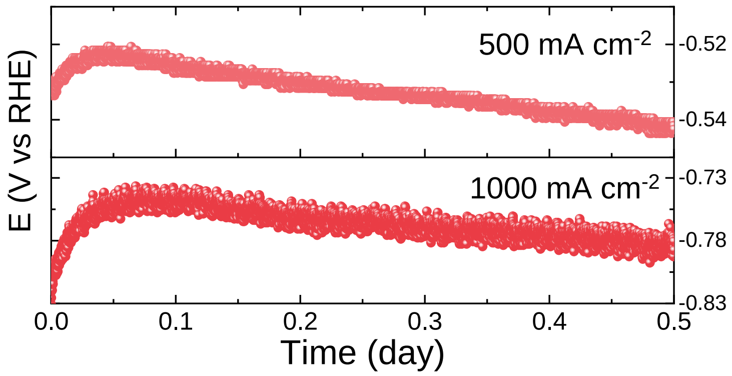

Supplementary Fig. 27 | HER performance in the initial period of long-term tests. The performance change results from surface reconstruction in the beginning several hours.

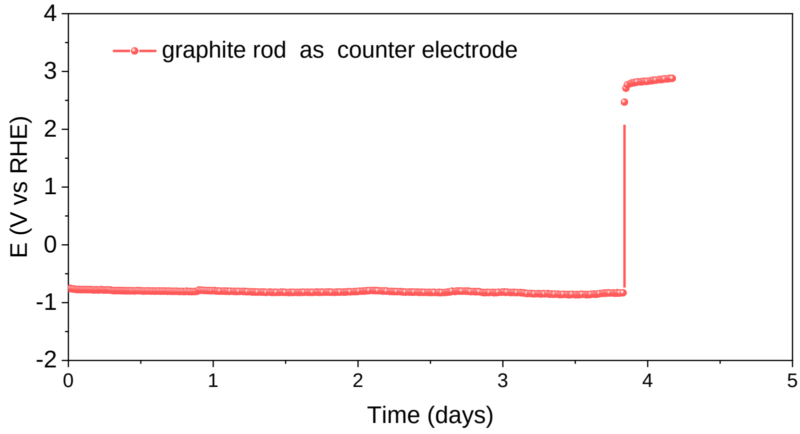

Supplementary Fig. 28 | Long-term stability at 1000 mA cm^{-2}. It was measured using a graphite rod as the counter electrode in 1M KOH.

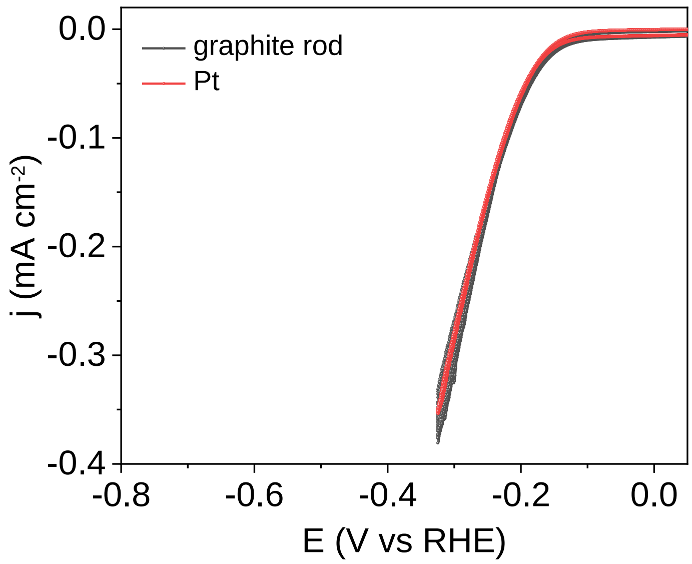

Supplementary Fig. 29 | CV curves. The curves were measured using a graphite counter rod for the first 50 cycles and then a Pt counter electrode for the other 50 cycles.

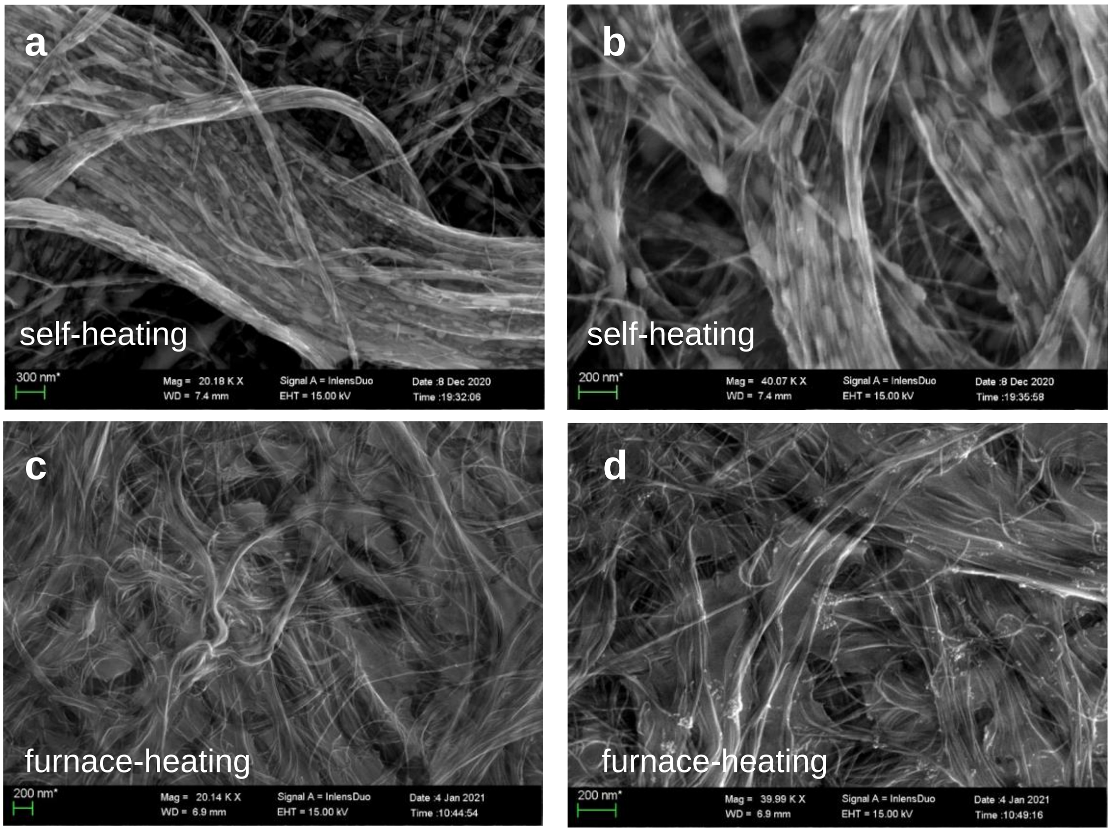

Supplementary Fig. 30 | SEM images of self-heating and furnace-heating samples. **a,b** Self-heating (Mo_2C/MoC/CNT) film. **c,d** Furnace-heating (f-Mo_2C/MoC/CNT) film. In the Mo_2C/MoC/CNT film, Mo_2C/MoC exists as small particles which serve as solder joints to strengthen CNT substrate and combine with CNTs firmly, while in the f-Mo_2C/MoC/CNT film, Mo_2C/MoC exists as large bulks with poor crystallinity.

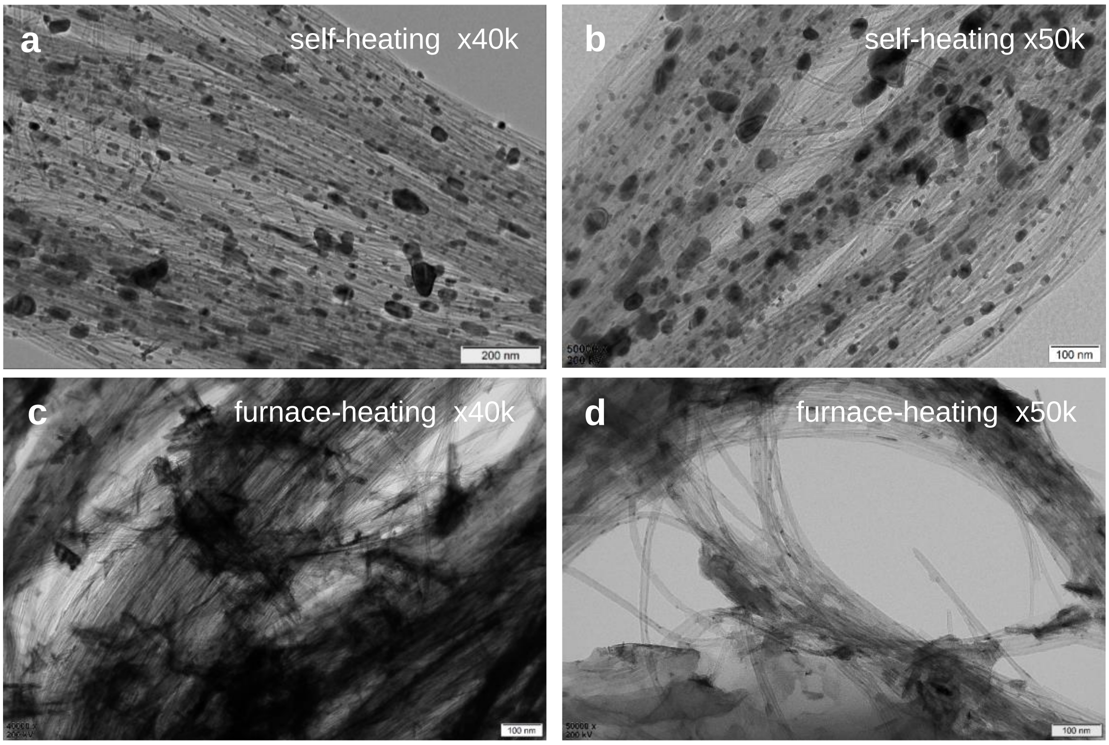

Supplementary Fig. 31 | TEM images of self-heating and furnace-heating samples.

a,b Self-heating (Mo_2C/MoC/CNT) film. **c,d** Furnace-heating (f-Mo_2C/MoC/CNT) film.

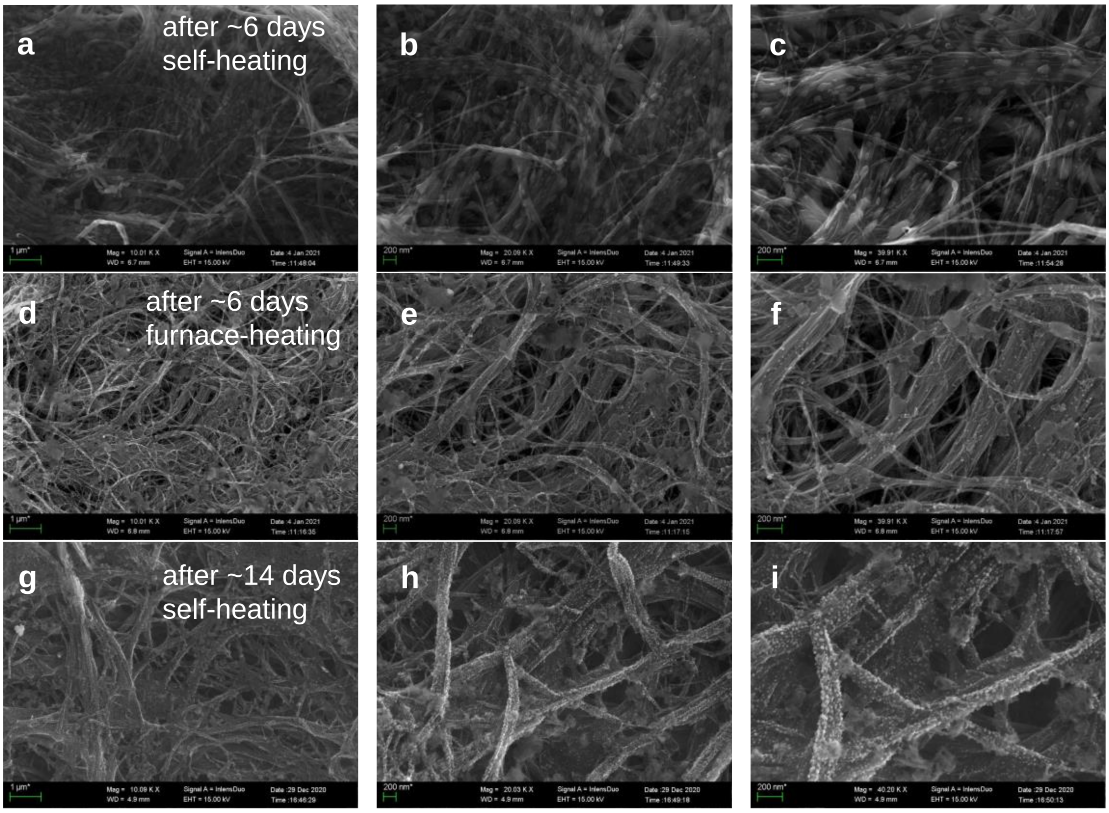

Supplementary Fig. 32 | SEM characterization of samples after long-term stability tests. a-c SEM images of Mo_2C/MoC/CNT film after working at 1000 mA cm^{-2} for ~6 days. **d-f** SEM images of f-Mo_2C/MoC/CNT film after working at 1000 mA cm^{-2} for ~6 days. **g-i** SEM images of Mo_2C/MoC/CNT film after working at 1000 mA cm^{-2} for ~14 days.

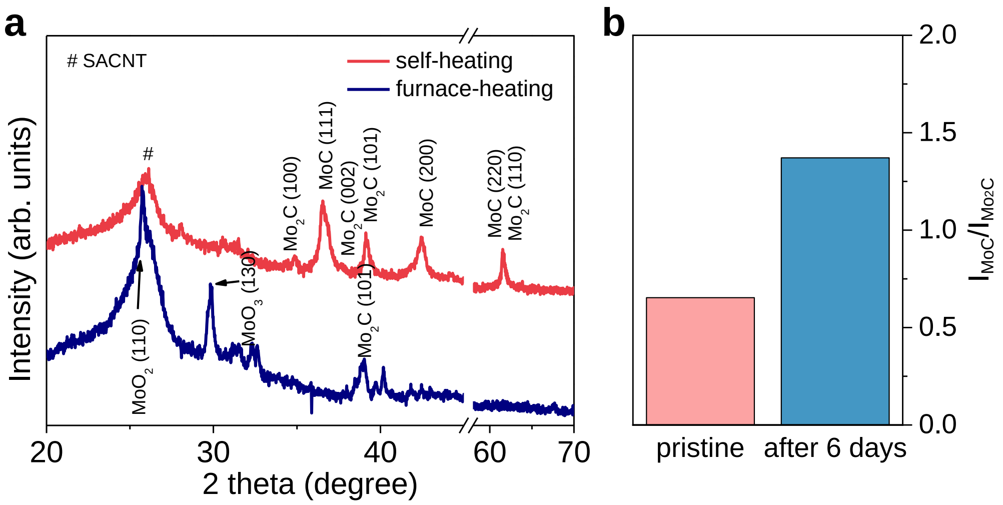

Supplementary Fig. 33 | XRD analysis after long-term stability tests. a XRD spectra of Mo_2C/MoC/CNT film and f-Mo_2C/MoC/CNT film after working at 1000 mA cm^{-2} for ~6 days. **b** Peak intensity ratio of MoC (111) to Mo_2C (101) changes from ~0.7 for the pristine Mo_2C/MoC/CNT film to ~1.4 for the Mo_2C/MoC/CNT film working at 1000 mA cm^{-2} for ~6 days.

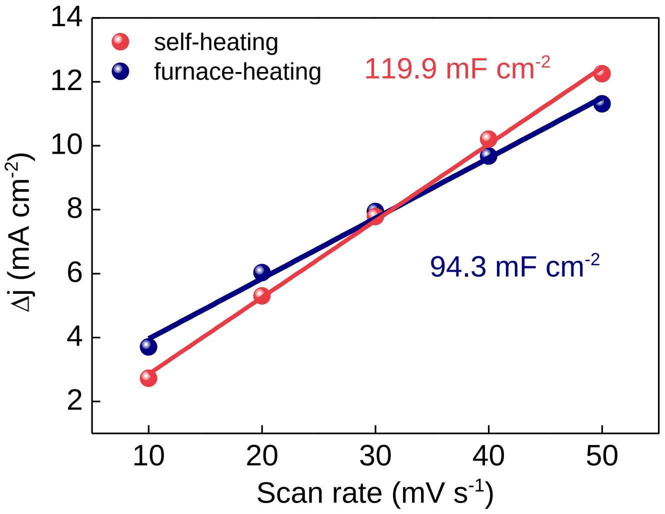

Supplementary Fig. 34 | Electrochemical C_{dl} of Mo_2C/MoC/CNT film and f-Mo_2C/MoC/CNT film. The data were measured in 1M KOH.

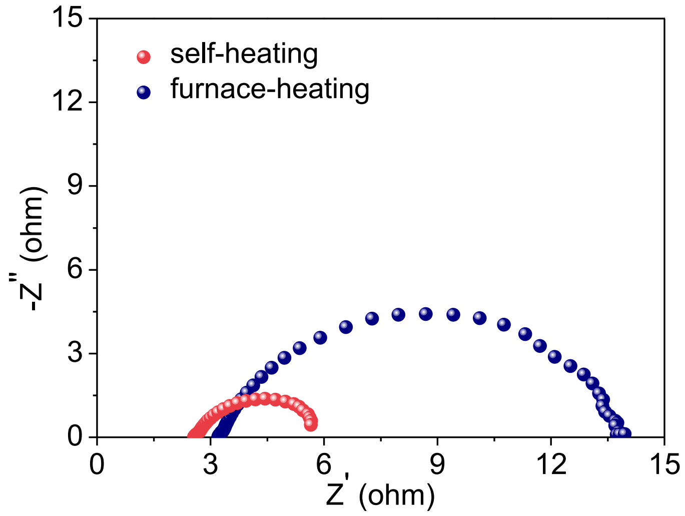

Supplementary Fig. 35 | Nyquist plots of Mo_2C/MoC/CNT film and f-Mo_2C/MoC/CNT film. The plots were measured at a potential of -150 mV versus RHE in 1M KOH.

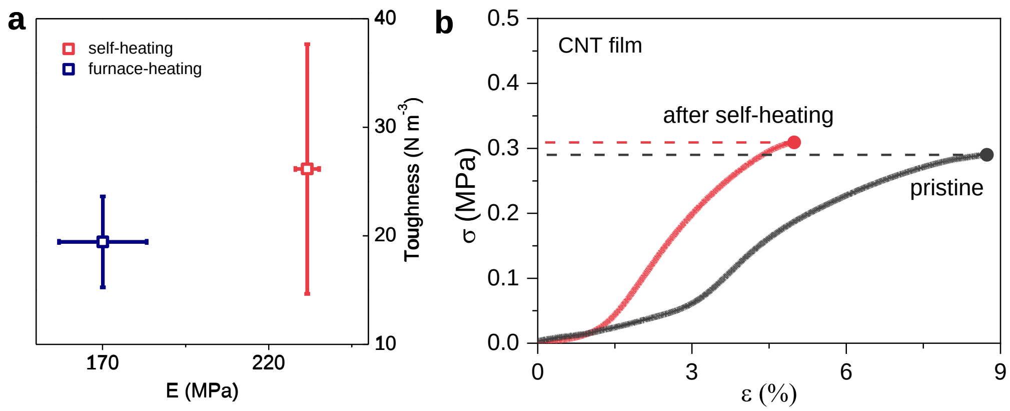

Supplementary Fig. 36 | Mechanical properties. a Young's modulus (E) and toughness of Mo_2C/MoC/CNT film and f-Mo_2C/MoC/CNT film. **b** Stress-strain curves of a pure CNT film before and after a self-heating process (30W for 30s and then 135W for 45s).

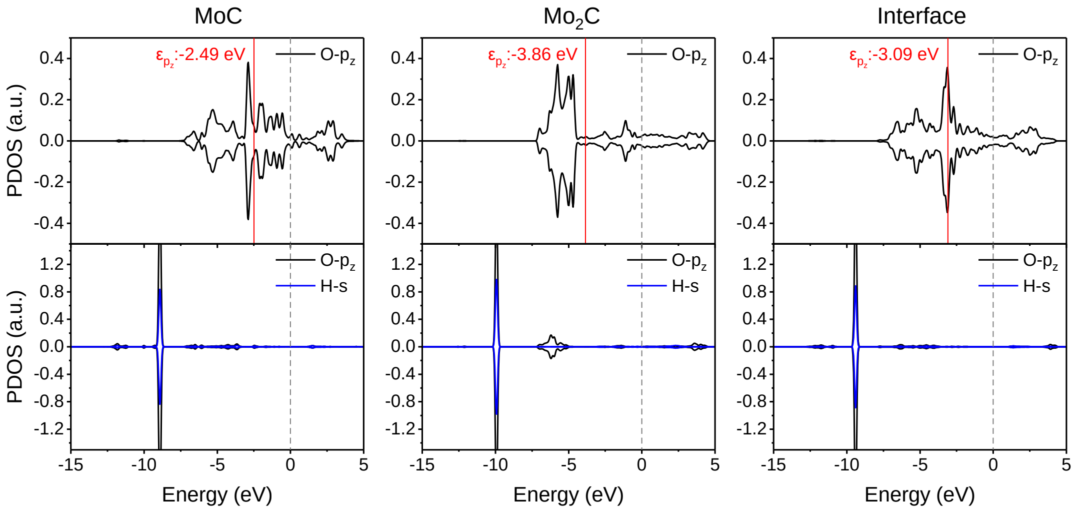

Supplementary Fig. 37 | Projected density of states (PDOS) analysis. PDOS of O-p_z orbital of O atoms adsorbed on MoC (111) surface, Mo_2C (100) surface, and Mo_2C/MoC interface before and after H adsorption. The solid red line indicates the p_z-band energy center (ε_{p_z}) of O atoms. The Fermi level is set to 0 eV.

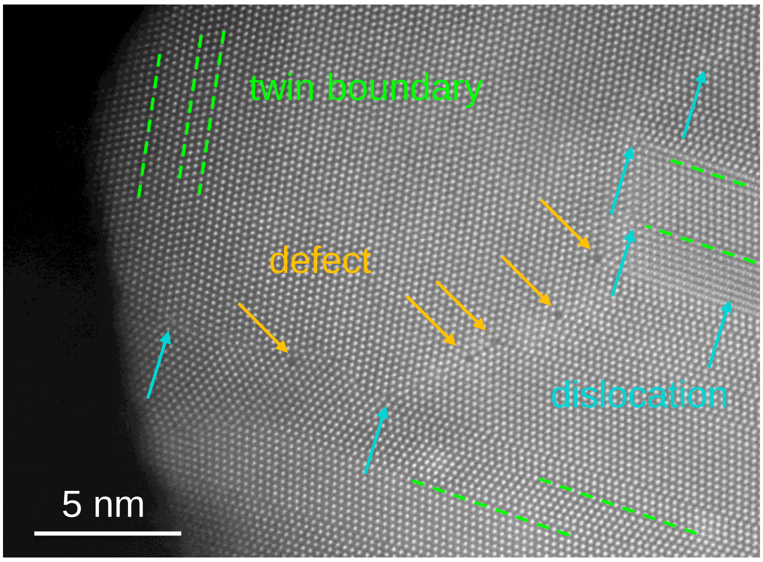

Supplementary Fig. 38 | HAADF-STEM image of Mo_xC. It shows that the material contains defects, dislocations, and twin boundaries.

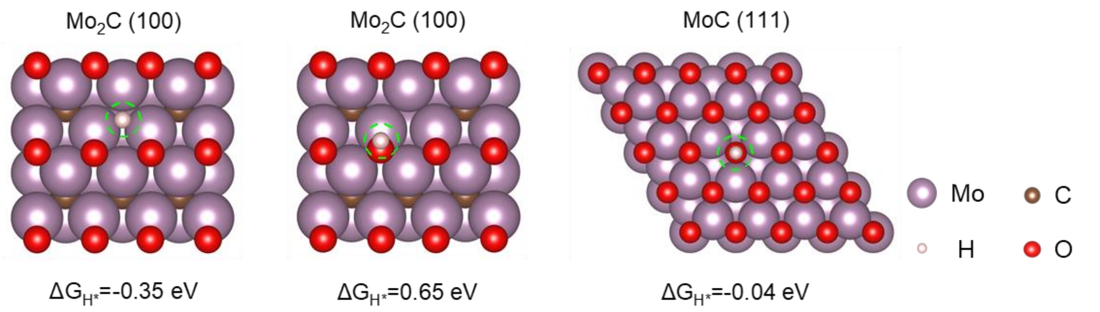

Supplementary Fig. 39 | DFT calculations of hydrogen adsorption. It shows adsorption structures and ΔG_{H*} of hydrogen on the Mo_2C (100) and MoC (111) surface with the carbon vacancy.

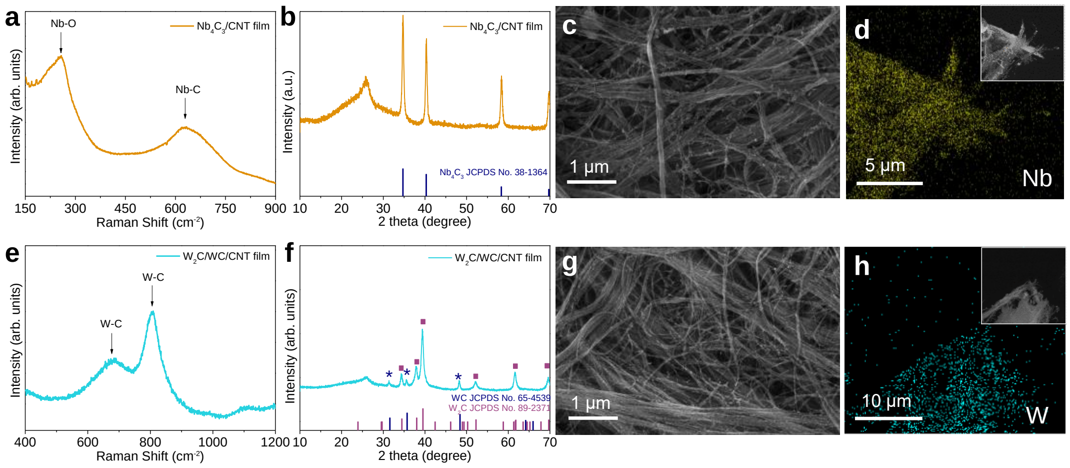

Supplementary Fig. 40 | Structural characterizations of different carbides/CNT films. a Raman spectra of a Nb_4C_3/CNT film. **b** XRD pattern of the Nb_4C_3/CNT film. **c** SEM image of the Nb_4C_3/CNT film. **d** EDS mapping of the Nb element in the Nb_4C_3/CNT film and the corresponding SEM image (inset). **e** Raman spectra of a W_2C/WC/CNT film. **f** XRD pattern of the W_2C/WC/CNT film. **g** SEM image of the W_2C/WC/CNT film. **h** EDS mapping of the W element in the W_2C/WC/CNT film and the corresponding SEM image (inset).

$C_4H_4NNbO_9 \cdot xH_2O$ and $(NH_4)_6H_2W_{12}O_{40} \cdot xH_2O$ serve as metal precursors for

NbC/CNT film and W_2C/WC/CNT film respectively, all with $C_6H_{12}O_6$ as carbon sources. Metal precursor and $C_6H_{12}O_6$ are added in a mixed solution of water and ethanol with a proportion of M:C = 4:3 (M= Nb, W). The precursor load on CNT film by dip coating of the mixed solution. Then precursor/CNT film is put into a quartz tube with 10% H_2 and 90% Ar for self-heating. We adopt same heating parameters as Mo_2C/MoC/CNT film of 30s at 30W and 45s at 135W to simply verify the feasibility of our method. Consequently, Nb_4C_3/CNT film, and W_2C/WC/CNT film are synthesized efficiently only with a different precursor, as shown in the Raman spectra and XRD patterns.[5-7] All of the hybrid CNT films have uniform particle dispersion and similar morphology as Mo_2C/MoC/CNT film. Whether a simple substance or a single-phase/composite compound will be synthesized by self-heating method and we might optimize the heterostructure by adjusting other parameters like heating time and output power.

Supplementary Tab. 1 | Mass percentage of N, C, and H elements measured by the materials element analyser.

Sample Name	(N) %	(C) %	(H) %
w/o urea-1#	0.000	59.267	0.192
w/o urea-2#	0.000	62.797	0.000
w/ urea-1#	0.000	51.131	0.000
w/ urea-2#	0.000	44.392	0.599

Supplementary Tab. 2 | Comparison of our Mo_2C/MoC/CNT catalysts with other HER catalysts working at large current densities in literature. The synthesis time and productivity (synthesis cycles in a day) exclude the pre-/post-processing time of HER catalysts.

Catalyst	Overpotential @1000 mA cm^{-2}	synthesis time (h)	Productivity (synthesis cycles per day)	Ref.
Mo_2C/MoC/CNT film	233	0.0125	1920	This work
IrFe/NC	850	3	8	[8]
MoS_2/Mo_2C	220	2	12	[9]
2H-$Nb_{1.35}S_2$	~370	1	24	[3]
$Ni_{2(1-x)}Mo_{2x}P$	294	1	24	[10]
MoS_2/Ni_3S_2	200	13.3	1.8	[11]
Ni_2P-Fe_2P	389	1.5	16	[12]
Sn–Ni_3S_2	570	5	4.8	[13]

MoS_2/Mo_2C	440	2.17	11.1	[14]
α-MoB_2	334	0.25	96	[15]
LiCoBPO/NF	400	80	0.3	[16]
Ni_2P/NF	306	0.5	48	[17]
NiCo LDH/NF	381	34.3	0.7	[18]

*The synthesis time only refers to the time consuming during key processes here, without the time such as heating, pre-synthesis processing, and post-cooling. In our method, the pre-synthesis processing includes CNT film preparation, laser drilling, and precursor loading, which takes about 15 minutes in total. After a self-heating synthesis at high temperature, the as-prepared composite CNT film can be served as an electrode directly. Our method costs a comparable pre-/post-synthesis processing time and a much shorter synthesis time than traditional methods.

Supplementary Tab. 3 | Comparison of stability after CV cycles of our samples and other HER catalysts.

Catalyst	Cycles	Current density (mA cm^{-2})	△overpotential (mV)	Ref.
Mo_2C/MoC/CNT film	10000	0-1500	<10	This work
IrFe/NC	10000	200	20	[8]
$Ni_{12}P_5$-$Ni_4Nb_5P_4$	3000	100	16	[19]
MoS_2/Mo_2C	10000	120	11	[9]
1T–MoS_2 /$Ni(OH)_2$	1000	500	5	[20]
MoP	1000	100	23	[21]
Ni_3N-NiMoN	5000	200	19	[22]
MoP@RGO	5000	20	23	[23]
$Cr_{0.4}Mo_{0.6}B_2$	5000	200	10	[24]
MoS_2	5000	10	5	[25]
MoS_2−Ni_3S_2	10000	100	22	[26]
Ni NP|Ni–N–C	2000	50	16	[27]

*The underlined data is measured from the corresponding literature.

Supplementary Tab. 4 | ICP result of the electrolyte after a long-term stability test for 14 days.

Samples	Mo	Pt	Dimension
Mo_2C/MoC/CNT film	5.05	< 0.1	μg/mL

Supplementary Tab. 5 | Comparison of long-term stability among our samples and other HER catalysts.

Catalyst	Current density (mA cm^{-2})	Time (hours)	△overpotential (mV)	C_{sta}	Ref.
Mo_2C/MoC/ CNT film	500<br>1000	336<br>336	32<br>47	$1.89x10^7$<br>$2.57\ x10^7$	This work
MoS_2/Ni_3S_2	1000	12	33	$1.31x10^6$	[11]
NiCo/NiCo −OH	500	24	55	$7.85x10^5$	[28]
1T–MoS_2 /$Ni(OH)_2$	500	100	37	$4.86x10^6$	[20]
Ni_2P-Fe_2P	100(24h) &500(24h)	48	73	$7.10x10^5$	[12]
NFN-MOF/NF	500	30	14.65	$3.69x10^6$	[29]
IrFe/NC	200	0.5	32.7	$1.10x10^4$	[8]
HEI	100	40	34	$4.24x10^5$	[30]
CoNi/$CoFe_2O_4$/Ni	100	48	5.56	$3.11x10^6$	[31]
Co_3O_4	100	20	3.3	$2.18x10^6$	[32]
$MoNi_4$/MnO_2	10(10h) &100(10h) &200(10h)	30	11.8	$9.46x10^5$	[33]
Fe-(NiS_2/MoS_2)/CNT	10(8h) &100(8h) &200(8h)	24	27	$3.31x10^5$	[1]
CoP/NiCoP/	10(26h)	80	17.2	$4.19x10^5$	[34]

NC	&50(30h) &10(24h)				
Co-NiS_2	10,20,50,100	90	33	$4.73x10^5$	[35]
Single atom NiI	20	24	95	$1.82x10^4$	[36]
NiSe/NF	50	12	4.58	$4.72x10^5$	[37]
MoS_x@NiO	10	13	42	$1.11x10^4$	[38]
S-MoS_2@C	10	24	40	$2.16x10^4$	[39]
FeIr/NF	10	100	181	$1.99x10^4$	[40]
NiFeP/SG	10	30	21.1	$5.12x10^4$	[41]
$M_{o2}CT_x$/2H-MoS_2	10	240	10	$8.64x10^5$	[42]

*The underlined data is measured from the corresponding literature.

Supplementary Tab. 6 | Vacancy formation energies of Mo or C in Mo_2C and MoC surfaces.

Surface type	Mo vacancy (eV)	C vacancy (eV)
Mo_2C	2.71	0.35
MoC	1.87	-1.39

Supplementary References

1. Li C, *et al.* A lightly Fe-doped (NiS_2/MoS_2)/carbon nanotube hybrid electrocatalyst film with laser-drilled micropores for stabilized overall water splitting and pH-universal hydrogen evolution reaction. *J Mater Chem A* **8**, 17527-17536 (2020).

2. Liu D, *et al.* Atomically dispersed platinum supported on curved carbon supports for efficient electrocatalytic hydrogen evolution. *Nat Energy* **4**, 512-518 (2019).

3. Yang J, *et al.* Ultrahigh-current-density niobium disulfide catalysts for hydrogen evolution. *Nat Mater* **18**, 1309-1314 (2019).

4. Guan D, *et al.* Screening highly active perovskites for hydrogen-evolving reaction via unifying ionic electronegativity descriptor. *Nat Commun* **10**, 3755 (2019).

5. Coy E, *et al.* High Electrocatalytic Response of a Mechanically Enhanced NbC Nanocomposite Electrode Toward Hydrogen Evolution Reaction. *ACS Appl Mater Interfaces* **9**, 30872-30879 (2017).

6. Zhao S, *et al.* Li-ion uptake and increase in interlayer spacing of Nb_4C_3 MXene. *Energy Storage Mater* **8**, 42-48 (2017).

7. Ma Y-Y, *et al.* Highly efficient hydrogen evolution triggered by a multi-interfacial Ni/WC hybrid electrocatalyst. *Energy Environ Sci* **11**, 2114-2123 (2018).

8. Jiang P, *et al.* Improving electrocatalytic activity of iridium for hydrogen evolution at high current densities above 1000 mA cm^{-2}. *Appl Catal B* **258**, 117965 (2019).

9. Luo Y, *et al.* Morphology and surface chemistry engineering toward pH-universal catalysts for hydrogen evolution at high current density. *Nat Commun* **10**, 269 (2019).

10. Yu L, *et al.* Ternary $Ni_{2(1-x)}Mo_{2x}P$ nanowire arrays toward efficient and stable

hydrogen evolution electrocatalysis under large-current-density. *Nano Energy* **53**, 492-500 (2018).

11. Xue S, Liu Z, Ma C, Cheng H-M, Ren W. A highly active and durable electrocatalyst for large current density hydrogen evolution reaction. *Sci Bull* **65**, 123-130 (2020).

12. Wu L*, et al.* Heterogeneous Bimetallic Phosphide Ni_2P - Fe_2P as an Efficient Bifunctional Catalyst for Water/Seawater Splitting. *Adv Funct Mater* **31**, 2006484 (2020).

13. Jian J*, et al.* Sn-Ni_3S_2 Ultrathin Nanosheets as Efficient Bifunctional Water-Splitting Catalysts with a Large Current Density and Low Overpotential. *ACS Appl Mater Interfaces* **10**, 40568-40576 (2018).

14. Zhang C*, et al.* High-throughput production of cheap mineral-based two-dimensional electrocatalysts for high-current-density hydrogen evolution. *Nat Commun* **11**, 3724 (2020).

15. Chen Y*, et al.* Highly Active, Nonprecious Electrocatalyst Comprising Borophene Subunits for the Hydrogen Evolution Reaction. *J Am Chem Soc* **139**, 12370-12373 (2017).

16. Menezes PW*, et al.* Helical cobalt borophosphates to master durable overall water-splitting. *Energy Environ Sci* **12**, 988-999 (2019).

17. Yu X*, et al.* "Superaerophobic" Nickel Phosphide Nanoarray Catalyst for Efficient Hydrogen Evolution at Ultrahigh Current Densities. *J Am Chem Soc* **141**, 7537-7543 (2019).

18. Yang H, Chen Z, Guo P, Fei B, Wu R. B-doping-induced amorphization of LDH for

large-current-density hydrogen evolution reaction. *Appl Catal B* **261**, 118240 (2020).

19. Chen D*, et al.* Mulberry-Inspired Nickel-Niobium Phosphide on Plasma-Defect-Engineered Carbon Support for High-Performance Hydrogen Evolution. *Small* **16**, e2004843 (2020).

20. Chen W*, et al.* Achieving Rich and Active Alkaline Hydrogen Evolution Heterostructures via Interface Engineering on 2D 1T‐MoS_2 Quantum Sheets. *Adv Funct Mater* **30**, 2000551 (2020).

21. Song H, Li Y, Shang L, Tang Z, Zhang T, Lu S. Designed controllable nitrogen-doped carbon-dots-loaded MoP nanoparticles for boosting hydrogen evolution reaction in alkaline medium. *Nano Energy* **72**, 104730 (2020).

22. Wu A*, et al.* Integrating the active OER and HER components as the heterostructures for the efficient overall water splitting. *Nano Energy* **44**, 353-363 (2018).

23. Zhang G, Wang G, Liu Y, Liu H, Qu J, Li J. Highly Active and Stable Catalysts of Phytic Acid-Derivative Transition Metal Phosphides for Full Water Splitting. *J Am Chem Soc* **138**, 14686-14693 (2016).

24. Park H, Lee E, Lei M, Joo H, Coh S, Fokwa BPT. Canonic-Like HER Activity of $Cr_{1-x}Mo_xB_2$ Solid Solution: Overpowering Pt/C at High Current Density. *Adv Mater* **32**, e2000855 (2020).

25. Anjum MAR, Jeong HY, Lee MH, Shin HS, Lee JS. Efficient Hydrogen Evolution Reaction Catalysis in Alkaline Media by All-in-One MoS2 with Multifunctional Active Sites. *Adv Mater* **30**, e1707105 (2018).

26. Yang Y*, et al.* MoS_2–Ni_3S_2 Heteronanorods as Efficient and Stable Bifunctional Electrocatalysts for Overall Water Splitting. *ACS Catal* **7**, 2357-2366 (2017).

27. Lei C*, et al.* Efficient alkaline hydrogen evolution on atomically dispersed Ni–N_x Species anchored porous carbon with embedded Ni nanoparticles by accelerating water dissociation kinetics. *Energy Environ Sci* **12**, 149-156 (2019).

28. Zhu W*, et al.* NiCo/NiCo–OH and NiFe/NiFe–OH core shell nanostructures for water splitting electrocatalysis at large currents. *Appl Catal B* **278**, 119326 (2020).

29. Senthil Raja D, Chuah X-F, Lu S-Y. In Situ Grown Bimetallic MOF-Based Composite as Highly Efficient Bifunctional Electrocatalyst for Overall Water Splitting with Ultrastability at High Current Densities. *Adv Energy Mater* **8**, 1801065 (2018).

30. Jia Z*, et al.* A Novel Multinary Intermetallic as an Active Electrocatalyst for Hydrogen Evolution. *Adv Mater* **32**, e2000385 (2020).

31. Li S*, et al.* Bifunctional CoNi/$CoFe_2O_4$/Ni foam electrodes for efficient overall water splitting at a high current density. *J Mater Chem A* **6**, 19221-19230 (2018).

32. Zhang H, Zhang J, Li Y, Jiang H, Jiang H, Li C. Continuous oxygen vacancy engineering of the Co_3O_4 layer for an enhanced alkaline electrocatalytic hydrogen evolution reaction. *J Mater Chem A* **7**, 13506-13510 (2019).

33. Zhang J*, et al.* Efficient hydrogen production on $MoNi_4$ electrocatalysts with fast water dissociation kinetics. *Nat Commun* **8**, 15437 (2017).

34. Boppella R, Tan J, Yang W, Moon J. Homologous CoP/NiCoP Heterostructure on N‐Doped Carbon for Highly Efficient and pH‐Universal Hydrogen Evolution Electrocatalysis. *Adv Funct Mater* **29**, 1807976 (2019).

35. Yin J, *et al.* Atomic Arrangement in Metal-Doped NiS_2 Boosts the Hydrogen Evolution Reaction in Alkaline Media. *Angew Chem Int Ed* **58**, 18676-18682 (2019).

36. Zhao Y, *et al.* Non-metal Single-Iodine-Atom Electrocatalysts for the Hydrogen Evolution Reaction. *Angew Chem Int Ed* **58**, 12252-12257 (2019).

37. Tang C, Cheng N, Pu Z, Xing W, Sun X. NiSe Nanowire Film Supported on Nickel Foam: An Efficient and Stable 3D Bifunctional Electrode for Full Water Splitting. *Angew Chem Int Ed* **127**, 9483-9487 (2015).

38. Ibupoto ZH, *et al.* MoS_x@NiO Composite Nanostructures: An Advanced Nonprecious Catalyst for Hydrogen Evolution Reaction in Alkaline Media. *Adv Funct Mater* **29**, 1807562 (2019).

39. Xu Q, Liu Y, Jiang H, Hu Y, Liu H, Li C. Unsaturated Sulfur Edge Engineering of Strongly Coupled MoS_2 Nanosheet-Carbon Macroporous Hybrid Catalyst for Enhanced Hydrogen Generation. *Adv Energy Mater* **9**, 1802553 (2019).

40. Shen F, *et al.* Bimetallic iron-iridium alloy nanoparticles supported on nickel foam as highly efficient and stable catalyst for overall water splitting at large current density. *Appl Catal B* **278**, 119327 (2020).

41. Li R-Q, *et al.* Monolithic electrode integrated of ultrathin NiFeP on 3D strutted graphene for bifunctionally efficient overall water splitting. *Nano Energy* **58**, 870-876 (2019).

42. Lim KRG, *et al.* 2H-MoS_2 on Mo_2CT_x MXene Nanohybrid for Efficient and Durable Electrocatalytic Hydrogen Evolution. *ACS Nano* **14**, 16140-16155 (2020).
